# The application of geographic information systems (GIS) in identifying the priority areas for maternal care and services

**DOI:** 10.1186/s12913-017-2423-9

**Published:** 2017-07-12

**Authors:** Fatemeh Salehi, Leila Ahmadian

**Affiliations:** 1 Health Information Technology, School of Management and Medical Information, Kerman University of Medical Sciences, Kerman, Iran; 2 Research Center for Modeling in Health, Institute for Futures Studies in Health, Kerman University of Medical Sciences, Kerman, Iran; 3 Medical Informatics, Medical Informatics Research Center, Institute for Futures Studies in Health, Kerman University of Medical Sciences, Haftbagh Highway, Kerman, 7616911313 Iran

**Keywords:** Maternal health services, Geographic information system, Iran

## Abstract

**Background:**

Improving maternal health is globally introduced as an important health priority. The purpose of this study is to identify the high priority areas which require more maternal health services in Kerman, Iran.

**Methods:**

This is a descriptive cross-sectional study, performed in 2015. The literatures were first explored in order to extract geographic indicators and sub indicators relevant to the maternal health. Data were collected by the use of a questionnaire designed on the basis of AHP (Analytic Hierarchy Process) method. The validity and reliability of the questionnaire were confirmed by three medical informatics experts and test-retest method, respectively. Data were analyzed by Expert Choice software in order to specify the weight and importance of each indicator. The information were then added to Geographic Information System (GIS) to analyze and create the related maps.

**Results:**

Women’s access to hospitals plays an important role in identifying high priority areas which need maternal care and services. More than half of the mothers in Kerman have a moderate level of access to maternal care services. There is an association between facilities that are provided for pregnant women and the existence of healthcare centers. Moreover, there is a negative correlation between maternal death and the number of facilities provided for medical care and services for pregnant women.

**Conclusions:**

The application of GIS provides us with the capability to identify high priority areas which need maternal care. According to current population policies in Iran and the probable increase in the fertility rate, it is wise to plan proper schedules to improve health care services for pregnant women in Kerman.

**Electronic supplementary material:**

The online version of this article (doi:10.1186/s12913-017-2423-9) contains supplementary material, which is available to authorized users.

## Background

Nowadays prenatal care is considered as one of the most important topics in preventive medicine. The aim of prenatal care is to achieve a safe pregnancy which leads to delivery of a healthy newborn [1]. Prenatal care plays an important role in the health of mothers, fetuses and consequently children. According to the World Health Organization (WHO) report, in low-income countries only 46% of women benefit from adequate perinatal care, while in many parts of the world the level of antenatal care has improved substantially. This report indicates that millions of childbirths are not performed by professionals such as midwives, nurses or physicians. A host of reasons including poverty, long distances to medical care centers, inadequate facilities and cultural factors prevent mothers from receiving proper prenatal care [2].

Furthermore, maternal mortality, which may be due to pregnancy or labor complications, is considered as one of the most important factors that affect the mortality rate of the population. Maternal mortality can be influenced by health care services provided to pregnant women, educational status of mothers and their families, rural roads conditions, access to emergency obstetric care, health care costs, communications systems, family incomes and many other factors. Access to efficient perinatal health care and awareness of these services may serve preventive measures against maternal and neonatal mortalities. This necessitates a powerful health care system to provide care services at any time and any place. Therefore, identifying the areas needing health care services is important to fairly distribute these services. Moreover, studies have shown that there is a significant association between the availability of health care resources such as skilled and educated health providers and sufficient hospital beds, and a decrease in maternal mortality [3].

Many of the previous studies regarding the use of GIS in maternal health focused on potential geographic access to care on the basis of the spatial distribution of health facilities [4–8]. Some investigated the impact of geographic access on mortality and care utilization [9–11]. Other related studies were conducted to model EmOC (emergency obstetric care) availability and accessibility coverage [12–14].

An effective health care system is the one with a powerful monitoring system which considers the needs of the vulnerable groups and identifies any progress or problem in the system immediately. One of the latest technologies which can be used to promote better health care, health policy and decision-making is GIS (Geographical Information System) which is a computerized system to collect, keep, analyze and display geographic information [15]. GIS is a useful tool which identifies regional disparities in order to employ more educated individuals and to exert more health facilities [16, 17].

Studies indicated that an unfair health system, an uneven distribution of human resources within the health care system, inappropriate health policies, ignoring women’s health issues and inappropriate distribution of facilities or budget have resulted in increased mortality rates in Iran and have reduced women’s quality of life in deprived areas [18, 19]. Determining the areas that need health care services can help health care authorities provide adequate facilities to improve patients’ health.

Although GIS are widely used in public health and maternal care, there is a lack of published data on more explicit use of GIS and design of geographic pattern in extensive detail through combining various layers, indicators and risk factors that may be associated with maternal health and outcomes. Therefore, in this study GIS was applied to identify the high priority areas which need maternal cares and services in Kerman.

## Methods

### Study area

The province of Kerman is the largest province in the southeast of Iran and it is one of the areas with the highest rate of maternal death [20].

### Study design

This descriptive cross sectional study was aimed at identifying the high priority areas for maternal care and services and it was carried out in three stages. To identify the high priority areas for maternal care and services, first articles, books, forms, standards and guidelines [21–25] were explored in order to extract indicators and sub indicators relevant to the maternal health. An extensive search was performed and relevant literature were retrieved and evaluated based on the inclusion criteria. Inclusion criteria were all environment and geographic variables that directly or indirectly affect maternal health. According to semantic fields variables are classified into indicators and sub indicators. In the next step, a questionnaire was designed and data elements were entered into the questionnaire (Additional file 1). Questionnaire was designed based on AHP (Analytic Hierarchy Process) method. In order for the aim of this study to be fulfilled, the indicators should be weights of importance. One of the best and most common method for this goal is AHP method. The AHP is a multi-criteria decision making approach that uses a multi-level hierarchical structure of objectives, criteria, sub criteria, and alternatives. The data are derived by using a set of pair wise comparisons. These comparisons are used to obtain the weights of importance of the decision criteria, and the relative performance measures of the alternatives in terms of each individual decision criterion [26].

**Fig. 1:**
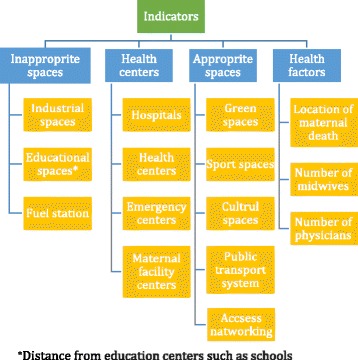
Indicators and sub indicators relevant to the mothers’ health

The questionnaires were completed by 15 experts in various field of health care who were related to maternal health. Fifteen individuals were recruited in this study 12 of whom were experts in health information technology, midwife, nurse, geography, expert in health family and health information management (a total of 12 people, consisting of two experts in each field) and three of whom were general practitioners and obstetricians. The experts that had experience working with pregnant women or their information in health care organization were included in study. This questionnaire consists of two parts. The first part consists of demographic characteristics of the participants such as age, gender and educational level. The second part contains four indicators and 15 sub indicators (Fig. 1). Questionnaire has 1–9 grades for each indicator. Content validity of questionnaire was evaluated and confirmed by 3 medical informatics specialists and one health information management expert through revision of content, relevance, grammar, wording, item allocation, and scaling. The reliability of the questionnaire was evaluated by a test-retest procedure on a sample of 7 experts; furthermore, the questionnaire achieved a Cronbach’s alpha of 0.8.

Expert Choice (version 11) was used to measure the relative weight of each indicator and sub indicator. Data inconsistency was calculated to be 0.04 using Expert Choice software. In the third stage, information related to the indicators were collected from various sources in order to identify the high priority regions for maternal care.

### Data resources

The information concerning maternal deaths and the number of human resources was obtained from the Health Deputy of Kerman University of Medical Sciences. Using 2009 Iran Census tract data, the distribution of reproductive age of women (15–49 years old) were identified. The population of reproductive women in this area was more than 70,000 in 2015. The locations of the remaining unresolved cases were confirmed through the district health offices by telephone contact. The facilities that do not offer maternity services were excluded. Information on other indicators such as industrial, educational and fuel stations places and network access (roads) was obtained from the Kerman Municipality. Then the data file created in Expert Choice was added to ArcGIS to analyze and create the related maps.

### GIS procedure

Locations providing health services to pregnant women including hospitals and health centers were geocoded using ArcGIS 10.0 (Environmental Systems Research Institute [ESRI], Redlands, California, United States). The facilities and addresses of home maternal mortality without latitude and longitude data were geo-referenced by manual matching of listed town names to mapped locations on Google Earth. All data were entered into GIS and converted from vector to raster layers. Then weightings of indicators and sub indicators that were specified by AHP method in raster layers were applied. In this study, we used Buffer tool in GIS to analyze the data. Geoprocessing is one of the most powerful components of a geographic information system (GIS). Geoprocessing allows you to define, manage, and analyze the information used to form decisions. Buffer is one of the geoprocessing and analysis tools that is commonly used in calculating proximity. This tool creates a new feature class of buffer polygons around either polygon, line, or point features [27].

### Ethical considerations

Regarding ethical considerations, patients’ information remained confidential and informed consent was obtained from their families. Ethical approval was received from the Kerman University of Medical Sciences. (Ethical number: IR.KMU.REC.1394.328).

## Results

**Fig. 2:**
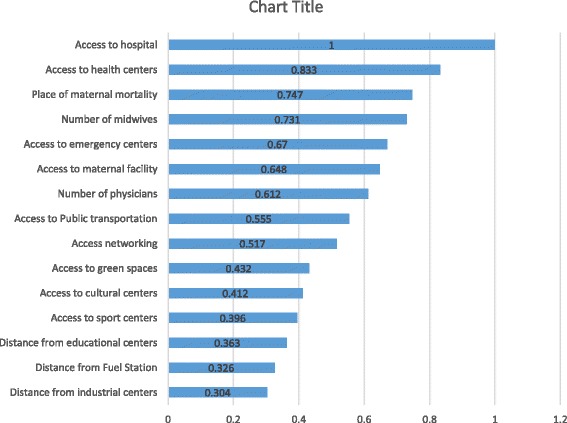
Values of indicators based on the analysis performed in Expert Choice software

The present study revealed that women’s access to hospitals plays an important role in identifying high priority areas which need maternal care and services related to the health of pregnant women. The base of this grading is the experts’ opinion. The analysis performed in this study revealed that women’s access to hospitals, access to healthcare centers and the places where pregnant women live, were graded as 1, 0.833 and 0.747, respectively. In addition, distance from industrial centers with the grade of 0.304 had a lower degree of importance (Fig. 2).

**Map 1:**
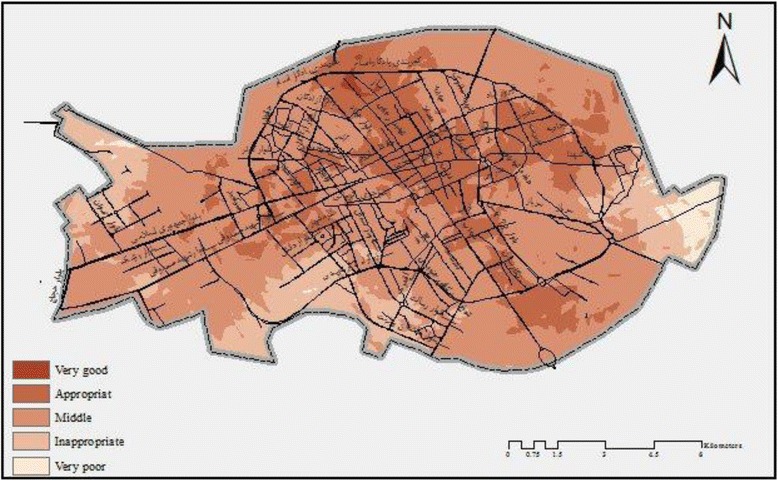
Kerman zoning- providing facilities for pregnant women at the time of this study

**Fig. 3:**
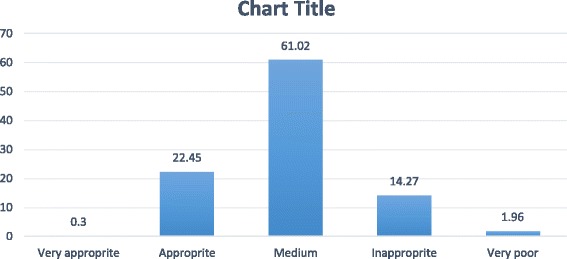
The quantity of facilities that are available in Kerman for pregnant women

The analytic hierarchy process was performed using the overly sum tool by Arc GIS application which is shown in Map 1. The results of the present study showed that services which were provided for mothers were average in 61.2% areas of Kerman. Although 22.45% of mothers had proper access to medical care facilities, 14.27% of them did not have access to acceptable facilities (Fig. 3).

Due to environmental features, the central zone of Kerman is superior in providing efficient facilities. However, the southern, eastern and western fringe areas have a lower rank in providing such facilities (Map 1).

**Map 2:**
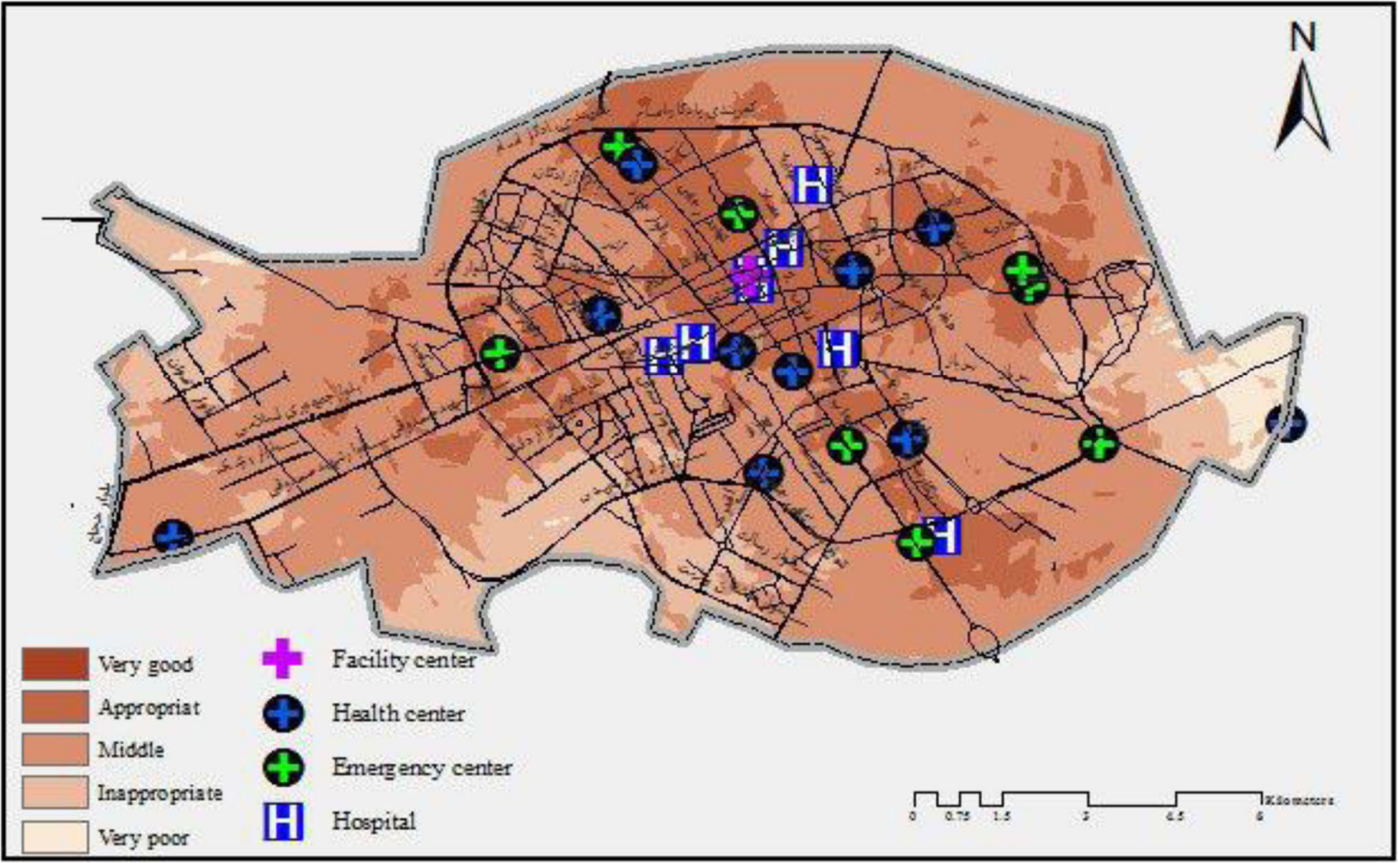
Spatial relationship between medical care and services for pregnant women in this study

**Map 3:**
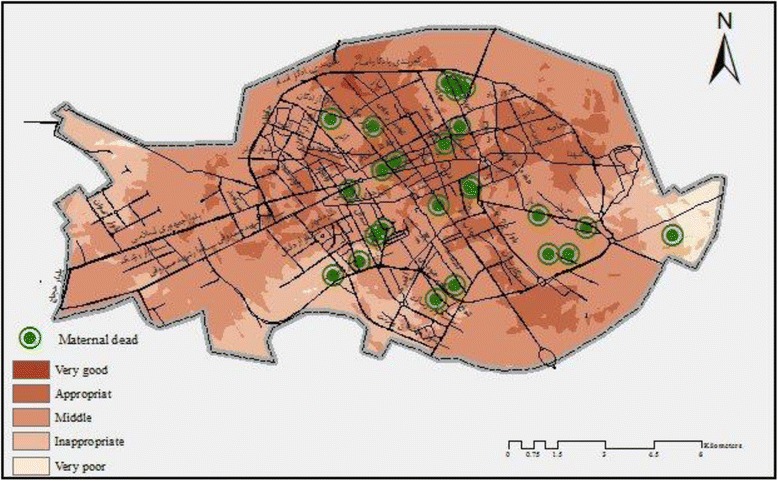
Spatial relationship between location of maternal death and services for pregnant women in this study

In addition, the present research study revealed that there is an association between facilities which are provided for medical care services for pregnant women and the existence of healthcare centers (Map 2). Therefore, when compared with the suburban areas, better medical care facilities are available in the center of the city of Kerman, since there are a large number of health care centers in that area. Furthermore, the spatial analysis of analytic hierarchy process showed that the majority of mortality rates were related to facilities which provided average quality medical care services. Also, the rate of maternal death was reported to be 16, 7 and 2 which occurred in areas with average, appropriate and inappropriate medical care facilities, respectively (Map 3).

Moreover, this study showed a negative correlation between the maternal death and the number of facilities provided for pregnant women.

## Discussion

This study was conducted to determine the high priority areas which need maternal care and services for pregnant women in Kerman in 2015. Our study revealed that women’s access to hospitals and health centers plays an important role in identifying such high priority areas which need maternal care and services. The result of this research study showed that more than half of the mothers in Kerman have access to moderate level services and there is an association between facilities provided for pregnant women and the existence of healthcare centers since better facilities and services have been provided in city center of Kerman.

The result of this study showed that there is associated correlation between maternal death and the number of facilities provided for pregnant women. This finding is supported by reports of various other authors who have noted that maternal mortality is associated with accesses to hospitals, health care centers and emergency obstetric care (EmOC) [7, 8, 12, 13, 28, 29]. Efforts to reduce maternal mortality by improving the quality, preparedness and availability of health care for pregnant women will have little impact where long distances, inadequate infrastructure, and poor transportation system mean that women are unable to physically access these services within a clinically appropriate timeframe [6]. We should also note that such activities may impart the wrong message by implying that only hospitals are useful in reducing maternal mortality [30]. This mistaken impression has given some policy makers the idea that reducing maternal deaths means building new hospitals and supplying them with sophisticated equipment and specialist physicians. This is not necessary in many places. On the other hand, it is not cost effective and it imposes high costs on the governments. We believe that one of the most effective means of intervention to reduce maternal mortality is the development of small and low-cost health centers in different areas, especially rural areas for pregnant women and women of reproductive age in order to provide them with easy access to these centers. With a trained midwife in these centers, mothers can be monitored on a regular basis and receive necessary pregnant care. In this case, they may be referred to hospitals to received special services in time if needed. Additionally, there is some evidence that integrating primary health services (or linkages) may improve the utilization and outcomes of healthcare delivery [31]. Integrating services help to bring together inputs, organization, and delivery of particular functions to increase efficiency and people’s access [32]. Findings from observational studies suggests that planned home birth is safe and may lead to fewer interventions, fewer complications and fewer neonatal problems [33, 34]. We also believe that according to result of this study and inadequate access of pregnant women to health centers, this plan will be beneficial.

To our knowledge, the current study is the first one using a Buffer tools to analyze the information of maternal health in GIS software. Most of studies used Hot spot analysis in GIS software [35, 36].

In the current study, women’s access to hospitals and health centers plays an important role in identifying such high priority areas which need maternal care and services. Similarly, the findings of previous studies pinpointed distance (or travelling time) to health care facilities as one of the major barriers to health care use, more especially in rural South Africa, where health care centers are often located in a further distance from a large number of residents [37, 38].

The results of this study showed that the outlying areas of Kerman city received poorer services than the central parts of the city. It is well known that the people living in remote areas, in general, are underprivileged when it comes to economic and educational facilities. Therefore, the authorities should pay more attention to these areas.

To the best of our knowledge, the majority of the studies just survey geographical access to health care [4–9] and a study that combines layers and mapping of various indicators related to geographic factors have not been reported so far.

### Limitations

There are several limitations to this study. First, although the results of this study underlines high priority areas to provide services and care to pregnant mothers, it does not offer any information about the quality of services that should be provided for pregnant mothers. Service availability is a prerequisite to quality services, but it does not guarantee the delivery of quality services. Additionally, data were obtained by the researchers in this study, however, they were collected within routine systems. Allocating resources appropriately will require collecting high-quality data that must be collected in a standard manner and be used routinely, timely and frequently at both the local level for improving the quality of programs and on the national scale for planning programs and allocating resources.

## Conclusion

Despite the rapid growth of technologies and health information systems, most of the health information systems do not merge patients’ records with external data sets. This can explain the reason why such isolated data systems cannot be used to recognize how the physical and environmental context of each patient influences his/her health choices and health outcomes. Therefore, this fact indicates the necessity of using tools such as GIS. Pregnant women access to health care centers and improvement in their health status are considered as basic rights of women and can be thought of as an index of development in any country. According to the current population policies in Iran, an increase in the fertility rate is expected in the near future. Therefore, it is wise to plan proper schedules to improve health care services for pregnant women. It is equally recommended to improve access of pregnant women to health care services since they are in need of immediate medical care.

Health policy makers and managers may be able to use the results of the present research study for auditing infrastructures, employing well trained staff to work in high priority areas, improving health care centers and clinics, reconstructing roads, and improving public transportation systems so that women have better access to health care centers.

## Additional file

Additional file 1: Questionnaire based on AHP method. Paired comparison of indicators and sub indicators regarding to fertility and midwifery services. The data are derived by using a set of pair wise comparisons. These comparisons are used to obtain the weights of importance of the decision criteria. (DOCX 36 kb)

## Abbreviations

EmOC: Emergency Obstetric Care
GIS: Geographic Information Systems
WHO: World Health Organization
